# Nutritional Value and Health Implications of Meat from Monogastric Animals Exposed to Heat Stress

**DOI:** 10.3390/nu17081390

**Published:** 2025-04-21

**Authors:** José A. M. Prates

**Affiliations:** 1 CIISA—Centro de Investigação Interdisciplinar em Sanidade Animal, Faculdade de Medicina Veterinária, Universidade de Lisboa, Av. da Universidade Técnica, 1300-477 Lisboa, Portugal; japrates@fmv.ulisboa.pt; 2 Associate Laboratory for Animal and Veterinary Sciences (AL4AnimalS), Av. da Universidade Técnica, 1300-477 Lisboa, Portugal

**Keywords:** climate change, heat stress, monogastric animals, meat quality, nutritional value, human health

## Abstract

Heat stress (HS), driven by rising global temperatures, significantly impairs the nutritional composition and sensory quality of meat from monogastric animals, particularly swine and poultry. HS induces physiological disturbances, including reduced feed intake, oxidative stress, and endocrine disruption, which together reduce muscle protein content by 10–15% and essential amino acid levels (e.g., lysine, methionine, threonine) by 15–25%. Lipid profiles are also altered, with up to 30% reductions in polyunsaturated fatty acids (PUFAs), especially omega-3s, and an increased saturated fat content. Additionally, HS reduces the retention of vitamins E, A, D, and C by 20–50% and critical minerals such as selenium, zinc, and iron, compromising antioxidant capacity, immune function, and oxygen transport. These changes diminish meat tenderness, juiciness, flavour, and colour stability, leading to reduced consumer appeal and dietary quality. The consumption of heat-stressed meat may elevate risks for cardiovascular disease, oxidative stress, and micronutrient deficiencies. Mitigation strategies, including dietary antioxidant and osmolyte supplementation, genetic selection for thermotolerance, and optimised feeding practices, can reduce oxidative damage by up to 40% and improve nutrient retention. This review synthesises the current evidence on HS-induced meat quality deterioration and explores nutritional and management strategies to protect animal productivity and human health.

## 1. Introduction

Climate change, characterized by increasing ambient temperatures and more frequent heat waves, significantly threatens global livestock production, especially in monogastric animals [1]. Understanding the impact of heat stress (HS) on these animals is thus essential for safeguarding food security and preserving meat quality [2]. The Intergovernmental Panel on Climate Change projects that global temperatures will rise by 1.5–2.0 °C by the end of the century, with more frequent and intense periods of HS expected to disrupt agricultural systems worldwide [3,4]. HS represents a significant threat to animal agriculture, particularly for monogastric species such as swine and poultry, which are highly sensitive to temperature fluctuations due to their limited capacity for thermoregulation through sweating and other physiological adaptations [5].

Monogastric animals represent a substantial portion of global meat production, accounting for approximately 77% of the total meat output, with poultry contributing 40% and swine 37% [5]. Monogastric animals are particularly vulnerable to HS due to their higher metabolic rates, limited sweating ability, and physiological constraints related to heat dissipation. While ruminants possess more complex digestive systems that may provide certain adaptive advantages, it is important to note they are also significantly affected by HS. Therefore, the relative susceptibility of monogastric animals should not overshadow HS’s considerable impacts on ruminant physiology and production. [3]. HS induces a cascade of metabolic and physiological disturbances that impair growth performance, nutrient metabolism, and meat quality. While recent studies have addressed various physiological responses to HS, important gaps remain, including the limited synthesis of molecular mechanisms directly linking HS to specific nutritional changes in meat, incomplete evaluations of nutritional mitigation strategies, and insufficient comprehensive analysis regarding subsequent implications for human health. Addressing these gaps is crucial for enhancing meat quality and safeguarding human nutrition in the context of climate change. However, precise economic impacts require further quantification [3].

HS triggers a complex cascade of physiological and metabolic disruptions in monogastric animals, beginning with increased body temperature and activation of the hypothalamic–pituitary–adrenal (HPA) axis [5]. The immediate physiological response includes an increased respiration rate, reduced feed intake, and increased blood flow to peripheral tissues to facilitate heat dissipation [6]. However, these adaptations come at a metabolic cost, reducing nutrient availability and impairing muscle development. Reducing feed intake under HS limits nutrient supply, particularly essential amino acids and fatty acids, which are critical for muscle protein synthesis and tissue repair [7].

Oxidative stress is a major consequence of HS in monogastric species. Elevated ambient temperatures lead to increased production of reactive oxygen species (ROS) in muscle tissues and other metabolically active organs, overwhelming the animal’s endogenous antioxidant defence systems [6]. Oxidative stress damages cellular components, including proteins, lipids, and DNA, impairing muscle metabolism and accelerating protein degradation. Furthermore, HS disrupts mitochondrial function, leading to impaired energy metabolism and reduced ATP production, which negatively impacts muscle growth and meat quality [8].

Endocrine and metabolic disturbances further compound these challenges. HS has been shown to alter circulating levels of key metabolic hormones, including insulin, leptin, and cortisol, which regulate appetite, energy balance, and nutrient partitioning [5]. Elevated cortisol levels promote muscle catabolism, while reduced insulin sensitivity limits glucose uptake and protein synthesis in muscle tissues. This endocrine imbalance results in decreased muscle mass, increased fat deposition, and altered fatty acid profiles, ultimately reducing the nutritional value and sensory quality of meat [7].

The physiological and metabolic disruptions caused by HS have direct and measurable effects on the quality and nutritional composition of meat from monogastric animals. Reduced muscle protein synthesis and increased protein degradation lead to lower muscle mass and diminished protein content in meat [9]. The fatty acid composition of meat is also adversely affected by HS. Elevated temperatures promote the accumulation of saturated fatty acids (SFAs) at the expense of polyunsaturated fatty acids (PUFAs), particularly omega-3 fatty acids, which are essential for cardiovascular and cognitive health in humans [8]. HS further compromises the retention of key vitamins and minerals in meat. Reduced feed intake and impaired nutrient absorption lower the bioavailability of fat-soluble vitamins (e.g., vitamins A, D, and E) and essential trace minerals (e.g., selenium and zinc) in muscle tissues [10].

This review synthesizes current scientific findings on the impact of HS on the nutritional value of meat from monogastric animals and its implications for human health. It also explores nutritional strategies to mitigate these effects, enhance animal welfare, and promote the economic sustainability of monogastric production systems under increasing environmental stress. To conduct a thorough review, a comprehensive search of the literature was performed using major databases PubMed (National Center for Biotechnology Information, Bethesda, MD, USA), Scopus (Elsevier, Amsterdam, Netherlands), and Web of Science (Clarivate, London, UK). The search strategy focused on keywords such as “heat stress”, “meat quality”, “nutritional value”, “monogastric physiology”, “nutritional interventions,” “poultry”, and “swine”. The collected articles were carefully evaluated, prioritizing studies published within the past decade to ensure relevance, identify current gaps, and propose integrated approaches for improving resilience to HS in monogastric animals.

## 2. Physiological Impact of Heat Stress on Monogastric Animals

Understanding HS in monogastric animals is crucial for linking its effects to meat quality and nutrition. Swine and poultry are particularly vulnerable due to their limited thermoregulation compared to ruminants, which rely on behavioural and metabolic adjustments [3]. Consequently, HS disrupts homeostasis, impairing growth, metabolism, and muscle development [11]. This section reviews the key physiological changes, including altered thermoregulatory responses, endocrine disruptions, mitochondrial function, oxidative stress, and nutrient utilization.

### 2.1. Thermoregulatory Mechanisms

Monogastric animals such as swine and poultry possess limited mechanisms for dissipating heat compared to other livestock species. In contrast, ruminants can sweat and engage in rumination, which, while producing metabolic heat, also enables adaptive changes in digestion and energy expenditure that aid in heat tolerance [12]. To cope with high ambient temperatures, monogastric animals increase peripheral blood flow by dilating skin blood vessels, thereby promoting heat loss through convection and radiation. However, this thermoregulatory mechanism incurs a metabolic cost, as increased peripheral blood flow diverts circulation from vital organs, particularly the gastrointestinal tract. Consequently, nutrient absorption and metabolic efficiency are impaired, ultimately reducing the availability of essential nutrients required for muscle growth and repair and contributing to suboptimal growth performance [13].

Poultry, in particular, exhibit panting as a primary thermoregulatory response. Panting promotes evaporative cooling through the respiratory tract, effectively lowering body temperature. However, this response leads to respiratory alkalosis, which disturbs the acid–base balance and may impair muscle metabolism and growth [12]. The increased respiratory rate under HS also increases energy expenditure, contributing to a negative energy balance and reducing overall performance.

Swine and poultry also exhibit behavioural adaptations such as reduced activity, seeking shade, and altering feeding patterns under HS. Reduced feed intake is a common response aimed at lowering metabolic heat production, but it also results in inadequate nutrient supply for muscle development and maintenance [14]. In swine, HS leads to increased lying time and a reduction in physical activity, further decreasing muscle tone and growth. In poultry, increased time spent panting and wing-lifting to increase airflow around the body also reduces feeding time, which directly limits nutrient intake and muscle accretion.

Another important behavioural adaptation seen under HS is the modification of feeding patterns. Animals experiencing HS tend to consume less feed during the hotter parts of the day and increase consumption during cooler periods. However, this disrupted feeding behaviour limits the availability of nutrients during the peak metabolic periods for muscle growth and protein synthesis. The cumulative effect of reduced nutrient intake and increased metabolic demands results in a negative energy balance, which contributes to poor growth performance and reduced meat quality [13].

While these thermoregulatory responses are essential for survival during acute heat events, they are energetically expensive. Chronic activation of these mechanisms under prolonged HS leads to a negative energy balance, reduced feed efficiency, and impaired muscle growth [15]. The redirection of blood flow toward peripheral tissues and away from the gut also increases the susceptibility of animals to gastrointestinal diseases and impairments in digestive function.

### 2.2. Endocrine and Metabolic Disruptions

HS profoundly affects the endocrine system, altering the secretion and activity of key metabolic hormones involved in growth, nutrient metabolism, and the stress response. The HPA axis is activated during HS, resulting in the release of glucocorticoids such as cortisol. Elevated cortisol levels, while beneficial for acute stress responses, have detrimental effects when chronically elevated [13].

Cortisol promotes proteolysis and lipolysis to provide energy under stress conditions. In monogastric animals, this catabolic state reduces muscle protein deposition and increases muscle degradation, thereby compromising overall meat quality [15].

The effect of HS on insulin sensitivity appears to vary across studies. While some research indicates that HS reduces insulin sensitivity, impairing glucose uptake and nutrient utilisation [12], other studies have reported increased insulin sensitivity, particularly in cases where animals reduce feed intake, leading to adaptive metabolic responses [3]. These contrasting findings highlight the complexity of HS responses and the need for further investigation.

Circulating thyroid hormones such as triiodothyronine (T3) and thyroxine (T4), which regulate basal metabolic rate, also decline under HS. Reduced thyroid hormone levels lower the overall metabolic rate and limit the animal’s capacity for muscle growth and tissue repair [13]. A reduced basal metabolic rate and lower thyroid hormone levels create an energy deficit, compounding the negative impact of HS on growth performance.

### 2.3. Muscle Metabolism and Oxidative Stress

HS also affects muscle metabolism and function. Mitochondria, the cellular powerhouses responsible for generating ATP through oxidative phosphorylation, are particularly sensitive to HS. Elevated temperatures impair mitochondrial membrane integrity and disrupt the electron transport chain, reducing ATP production and increasing electron leakage [16].

HS increases the production of ROS in muscle tissues. Excess ROS contribute to oxidative damage by causing lipid peroxidation, protein oxidation, and DNA damage. Lipid peroxidation alters the integrity of muscle cell membranes, leading to cell dysfunction and increased muscle degradation. Protein oxidation reduces the structural integrity of muscle fibres, contributing to toughness and decreased meat quality [15].

## 3. Nutritional Composition of Meat Under Heat Stress

HS disrupts the physiological balance of monogastric animals and alters the nutritional composition and quality of their meat. This adversely affects human health and food security, given meat’s role as a key source of proteins, essential fatty acids, vitamins, and minerals. The induced biochemical and structural modifications impair flavour, tenderness, juiciness, and shelf life. Understanding these impacts is vital for developing targeted nutritional and management strategies. This section focuses on how HS affects proteins, amino acids, fatty acids, vitamins, minerals, and key quality attributes like tenderness, juiciness, flavour, and colour stability.

### 3.1. Proteins and Amino Acids

One of the most significant nutritional changes induced by HS is the reduction in muscle protein content, which negatively impacts both animal performance and the nutritional adequacy of meat products. Under thermoneutral conditions, monogastric animal muscle typically contains 20–23 g of protein per 100 g of meat. However, exposure to HS can decrease this concentration by 10–15%, resulting in values as low as 17–20 g/100 g. This protein reduction is driven by a combination of impaired protein synthesis, increased proteolysis, reduced feed intake, and elevated systemic oxidative and metabolic stress [6,10]. In addition to the reduction in protein quantity, the structural integrity of muscle fibres is often compromised, altering water-holding capacity and postmortem pH decline, both of which affect final meat quality and shelf life.

Reduced feed intake during HS limits the availability of essential amino acids (EAAs), such as lysine, methionine, and threonine, which are fundamental for muscle protein accretion, structural maintenance, and immune responses. In standard pork and poultry meat, lysine concentrations typically range between 1.6 and 1.8 g/100 g, methionine between 0.5 and 0.6 g/100 g, and threonine between 0.8 and 1.0 g/100 g. These concentrations can decrease by 15–25% under HS, dropping lysine to as low as 1.3–1.5 g/100 g, methionine to 0.4–0.5 g/100 g, and threonine to 0.6–0.8 g/100 g. These reductions are exacerbated by compromised gastrointestinal absorption and enzyme activity, limiting nutrient uptake even further [17,18].

HS impairs muscle protein synthesis and enhances protein degradation through a dual mechanism. Under HS conditions, suppression of the mTOR/IGF-1 signalling cascade leads to a marked decrease in muscle protein synthesis and muscle accretion. Concurrently, HS triggers activation of the HPA axis, resulting in elevated cortisol levels. This cortisol surge further drives proteolytic processes by upregulating pathways such as the ubiquitin–proteasome system and autophagy, thereby accelerating muscle protein breakdown. Cortisol accelerates catabolic pathways by promoting the activity of proteolytic systems, including the ubiquitin–proteasome and autophagy–lysosome pathways [19]. These hormonal disruptions also reduce satellite cell proliferation, inhibiting muscle regeneration. Fast-growing breeds are especially affected due to elevated basal metabolic heat production and higher oxygen demand [20,21].

HS also induces qualitative shifts in amino acid profiles. A 10–20% reduction in the ratio of essential to non-essential amino acids (EAAs:NEAAs) has been reported in meat from heat-stressed animals. Glutamine and alanine concentrations can increase significantly; for example, glutamine may rise from 0.8 g/100 g to more than 1.1 g/100 g as a result of stress-induced proteolysis [18,22]. This shift reflects muscle catabolism and is associated with impaired amino acid transport, affecting the synthesis of proteins, enzymes, and even neurotransmitters in downstream consumers [23].

The selective degradation of myofibrillar proteins, particularly myosin and actin, which are rich in lysine, leucine, and isoleucine, further distorts amino acid composition. These changes undermine the protein quality of meat and affect its technological characteristics such as tenderness, chewiness, and suitability for processed meat products [24,25]. Flavour and aroma compounds derived from amino acid degradation are also altered, potentially resulting in less desirable sensory profiles.

Recent studies emphasize the importance of animal genetics and targeted dietary interventions in counteracting HS-induced muscle protein losses. For instance, dietary supplementation with antioxidants like vitamin E (up to 200 mg/kg feed) and organic selenium has been shown to reduce protein degradation and maintain muscle integrity, with observed preservation of up to 10% in muscle protein levels [26,27]. These interventions are more effective when integrated with polyphenols, betaine, or functional oils that provide cellular protection. Genetic lines overexpressing heat shock proteins (e.g., HSP70) and antioxidant enzymes have also demonstrated reduced oxidative muscle damage and better performance during thermal stress [28].

Quantitative assessments demonstrate that HS can reduce total muscle protein from 23 g/100 g to approximately 19.5 g/100 g. Specifically, lysine may drop from 1.8 to 1.4 g/100 g, methionine from 0.6 to 0.45 g/100 g, and threonine from 1.0 to 0.8 g/100 g. These biochemical shifts translate into decreased protein efficiency ratios, diminished amino acid digestibility, and lower bioavailability for human consumers, especially in nutritionally vulnerable populations [29,30]. Compounded with declining feed conversion rates and elevated mortality under HS, the economic and nutritional ramifications of protein loss are substantial [31].

In conclusion, HS severely compromises both the quantity and quality of muscle proteins through multifactorial mechanisms involving hormonal imbalances, oxidative stress, and inadequate nutrient intake. These effects ripple across the entire meat supply chain, influencing animal productivity, product quality, and human health. Preserving the protein integrity of meat under HS conditions will require a multifaceted strategy combining genetic selection for heat tolerance, optimized antioxidant-rich diets, and precise thermal environment management [32,33]. Future research should prioritize proteomic and metabolomic profiling of stressed muscle tissue to identify early biomarkers of degradation.

### 3.2. Lipids and Fatty Acid Profiles

HS induces significant disruptions in lipid metabolism, affecting carcass fat deposition and reducing the meat’s nutritional and technological quality. Lipids are crucial in cellular energy storage, membrane structure, and signalling processes. Under HS conditions, livestock exhibit altered lipid deposition patterns, increased oxidative stress, and deteriorated meat quality traits such as texture, flavour, and shelf life [3].

One of the most documented outcomes of HS is a shift in fatty acid composition, specifically, an increase in saturated fatty acids (SFAs) and a reduction in polyunsaturated fatty acids (PUFAs). In broiler chickens under thermoneutral conditions, meat typically contains 20–25% PUFAs, 35–40% monounsaturated fatty acids (MUFAs), and 30–35% SFAs. HS can reduce PUFAs by 15–30% while increasing SFAs by 10–20%, altering the PUFA/SFA ratio from the optimal range of 0.8–1.2 down to 0.5–0.7 [34].

At the biochemical level, HS increases reactive oxygen species (ROS) production, triggering lipid peroxidation and impairing the integrity of unsaturated fatty acids. This oxidative stress is coupled with altered gene expression involving enzymes like Δ9-desaturase and elongases, as well as transcription factors such as SREBP-1c and PPAR-α [35]. MDA levels, a key indicator of lipid peroxidation, can rise from 0.1–0.2 µmol/g to over 0.35 µmol/g in HS-exposed muscle tissues, suggesting an acceleration of oxidative damage and a decline in meat freshness and stability [22,34].

HS also disrupts hepatic lipid regulation. It promotes lipogenesis while inhibiting lipid oxidation due to dysregulation of key metabolic hormones such as insulin, glucocorticoids, and adiponectin [36]. These hormonal shifts upregulate the expression of lipogenic enzymes like acetyl-CoA carboxylase (ACC) and fatty acid synthase (FAS), contributing to increased fat deposition and lower lean meat yield [37].

From a technological perspective, elevated SFA and oxidative degradation of PUFAs impair the physical properties of meat. Lipid oxidation degrades colour stability, flavour, and water-holding capacity, leading to a shorter shelf life and reduced consumer appeal [38]. These defects are more pronounced in processed meats, where emulsification and fat stability are essential for product consistency. Additionally, the increased saturation of intramuscular fat results in firmer fat consistency, less favourable mouthfeel, and poor cooking performance due to altered melting behaviour.

Flavour and aroma are also negatively affected. HS-driven lipid oxidation leads to the formation of aldehydes and ketones, including hexanal and nonanal types, which impart rancid or metallic notes. These changes reduce consumer satisfaction and the marketability of the meat, even if other meat quality traits remain unchanged [39]. Moreover, HS may influence the Maillard reaction during cooking by altering precursor amino acid and lipid interactions, leading to suboptimal browning and aroma development.

Nutritional interventions offer a promising route to mitigate these effects. Supplementation with antioxidants such as vitamin E (up to 200 mg/kg feed), selenium (0.3–0.5 mg/kg), and polyphenols like grape seed extract has been shown to reduce MDA by 30–40% and improve fatty acid profiles [40,41]. Alpha-lipoic acid and selenomethionine also support mitochondrial health and antioxidant defence, reducing hepatic lipid accumulation. The use of functional oils, such as linseed oil or camelina oil, enriched with omega-3 fatty acids, may help restore the nutritional quality of meat under HS.

Genetic selection for heat-resilient breeds is another important avenue. Indigenous or naked-neck chicken lines and certain pig breeds maintain more stable lipid profiles and show reduced lipid peroxidation under HS conditions [28]. These traits are often associated with increased expression of HSP70 and antioxidant enzymes like glutathione peroxidase. Long-term breeding strategies targeting thermotolerance, efficient fat metabolism, and reduced oxidative susceptibility will be essential for sustainable meat production in warming climates.

Environmental and managerial approaches further support lipid preservation. Cooling strategies (e.g., fans, sprinklers), adjusted feeding times, and the use of stable lipid sources like coconut oil reduce metabolic heat production and oxidative pressure. These practices help sustain lipid digestibility and maintain meat quality even under high ambient temperatures [42]. Feeding animals during cooler parts of the day, adjusting dietary energy density, and avoiding oxidized fat ingredients are simple but effective strategies that producers can implement.

Advanced omics techniques, including lipidomics, metabolomics, and transcriptomics, are enabling deeper insight into how HS modulates lipid pathways. Studies show significant alterations in genes like PPARα and co-regulators of lipid oxidation, confirming their central role in heat-induced metabolic shifts [43]. These tools may soon allow for biomarker development and precision nutrition targeting HS-vulnerable metabolic processes. Integrating these approaches with real-time monitoring of thermal load could allow producers to implement proactive dietary or environmental modifications to safeguard lipid quality before irreversible damage occurs.

Summing up, HS significantly disrupts lipid metabolism in livestock, affecting the nutritional value, sensory characteristics, and processing quality of meat. These effects are driven by oxidative stress, hormonal dysregulation, and enzymatic reprogramming. A multifaceted approach, combining antioxidant-rich diets, heat-resilient genetics, omics-guided diagnostics, and climate-smart management, is essential to preserve lipid integrity and sustain meat quality amid a warming climate.

### 3.3. Vitamins and Minerals

HS significantly impairs the retention, metabolism, and bioavailability of vitamins and minerals in livestock muscle, micronutrients essential for maintaining muscle energy metabolism, immune function, antioxidant defence, and meat quality. Elevated ambient temperatures trigger oxidative stress and alter gastrointestinal function, reducing the uptake and storage of micronutrients. This not only weakens the physiological resilience of animals but also degrades the nutritional and sensory characteristics of meat, including colour stability, texture, and shelf life [9].

Fat-soluble vitamins (A, D, E, and K) are stored in lipid-rich tissues and are critical for antioxidant defence, immune responses, and membrane stability. HS depletes these vitamins by increasing oxidative stress and disturbing lipid metabolism. Vitamin E (α-tocopherol), a major antioxidant in muscle tissue, prevents lipid peroxidation by neutralizing ROS. However, during HS, ROS generation outpaces antioxidant defences, depleting vitamin E reserves and accelerating muscle degradation [10,44]. Vitamin E depletion results in increased MDA, impaired membrane integrity, increased drip loss, and off-flavours in meat. It also lowers the oxidative stability of muscle tissue, leading to rancidity and a shortened shelf life.

Vitamins A (retinol) and D (cholecalciferol) are also compromised under HS. Vitamin A supports vision, mucosal immunity, and epithelial cell integrity, while vitamin D regulates calcium–phosphorus homeostasis and bone metabolism. Studies show reduced feed intake and impaired intestinal absorption under HS diminish the plasma levels of both of these vitamins [45]. These reductions are not only due to decreased dietary intake but also heightened metabolic consumption to manage stress responses. Reduced availability contributes to immune suppression, compromised bone mineralization, and decreased meat yield and quality.

Vitamin K, though less studied, is affected indirectly. Its lipid-dependent absorption means that oxidative damage and altered fat metabolism under HS likely reduce its bioavailability. Since vitamin K supports coagulation and bone matrix formation, a decline may impair blood flow in muscle and reduce meat oxygenation, which can influence postmortem colour and stability [46]. As HS is associated with systemic inflammation and vascular stress, reduced vitamin K function may also contribute to tissue fragility and impaired healing, indirectly affecting muscle condition pre- and post-slaughter.

Water-soluble vitamins (B-complex and vitamin C) are especially vulnerable to HS due to their limited storage and high metabolic turnover. Vitamin C (ascorbic acid) is a key antioxidant and cofactor in collagen synthesis. Under HS, demand for vitamin C rises sharply, reducing its retention in muscle tissue [10]. This results in weakened ROS defence, impaired collagen formation, and reduced meat tenderness. Moreover, vitamin C enhances the regeneration of vitamin E and modulates iron metabolism, highlighting its central role in meat quality maintenance.

B-complex vitamins, including thiamine (B1), riboflavin (B2), pyridoxine (B6), and cobalamin (B12), play indispensable roles in energy metabolism and protein synthesis. HS suppresses appetite, elevates metabolic rate, and accelerates vitamin turnover, leading to depleted B-vitamin reserves in muscle [47]. This impairs mitochondrial ATP generation and amino acid processing, thereby weakening muscle function, regeneration, and the overall protein quality of meat. Additionally, B-vitamin deficiencies under HS conditions are linked to neuromuscular impairments and reduced red blood cell production, which further degrade muscle integrity.

Minerals are equally critical for muscle health, especially selenium (Se), zinc (Zn), iron (Fe), calcium (Ca), and magnesium (Mg). HS disturbs their balance by impairing absorption, increasing renal excretion, and shifting tissue distribution [48]. These disruptions compromise enzyme activities, reduce cellular defence against oxidative damage, and hinder muscle development and function.

Selenium is a cofactor in glutathione peroxidase that is crucial for antioxidant protection. HS increases selenium demand while simultaneously reducing its bioavailability. Selenium deficiency compromises meat shelf life by accelerating lipid oxidation and oxidative spoilage [49]. Supplementation with organic selenium has shown improvements in carcass oxidative stability and immune response in heat-exposed animals.

Zinc regulates hundreds of enzymes and is critical for immune modulation and antioxidant defence. HS leads to zinc loss via increased urinary excretion, thereby reducing SOD activity and impairing protein turnover [50]. This results in reduced muscle mass and diminished meat tenderness. Moreover, Zn influences DNA synthesis and apoptosis regulation, implicating it in tissue remodelling processes during thermal stress.

Iron plays a key role in myoglobin and haemoglobin synthesis. HS reduces iron uptake and increases metabolic turnover, leading to pale meat with lower myoglobin content and reduced colour stability [3]. Furthermore, oxidative stress can oxidize ferrous iron (Fe^2+^) to ferric iron (Fe^3+^), disrupting oxygen delivery within muscle cells and accelerating discoloration.

Calcium and magnesium are required for muscle contraction and cellular signalling. HS increases renal losses and reduces muscle uptake of both minerals, impairing contraction–relaxation cycles and leading to tougher meat and reduced consumer appeal [51]. Calcium is also essential for postmortem proteolytic activity, and its reduction can delay tenderization processes. Magnesium deficiency under HS is further associated with mitochondrial dysfunction, increased inflammation, and poor energy efficiency in muscle.

Collectively, these disruptions in vitamin and mineral metabolism under HS decrease meat quality through biochemical, sensory, and nutritional pathways. Targeted nutritional interventions, including dietary fortification with vitamins C, E, D, and trace minerals like Se and Zn, have shown promise in reducing these effects [52]. Future strategies may benefit from precision supplementation based on omics profiling to tailor nutrient support to specific environmental stress loads.

### 3.4. Meat Quality Attributes

HS significantly impairs key sensory attributes of meat, including tenderness, juiciness, flavour, and colour stability. These characteristics are crucial for consumer acceptance, economic valuation, and overall satisfaction with meat products. They are among the most important indicators of meat quality used by the food industry and consumers alike. The biochemical and structural alterations induced by HS negatively affect muscle protein integrity, lipid composition, pigment oxidation, and water-holding capacity. These changes reduce the palatability, shelf life, and nutritional value of meat [9].

Tenderness and juiciness, two critical components of eating quality, are mainly influenced by the structural state of muscle fibres, the rate of postmortem proteolysis, and the water-holding capacity (WHC) of muscle tissues. HS alters muscle metabolism and accelerates muscle protein breakdown through the activation of stress-responsive proteolytic pathways, including the calpain system and ubiquitin–proteasome system (UPS). These systems target essential contractile proteins such as actin, myosin, and titin, leading to faster degradation of the muscle fibre structure [53,54]. While this enzymatic activity can result in meat that is initially more tender, the structural damage reduces the ability of the muscle to retain water, which significantly compromises juiciness.

WHC, or the capacity of muscle to retain moisture during storage, cutting, and cooking, is a pivotal trait affected by HS. The oxidative damage incurred by muscle proteins impairs their functionality by altering amino acid side chains, particularly in proteins like myosin and actin that bind water. This leads to increased drip loss, greater cooking loss, and a drier texture in the final meat product [25]. Moreover, HS causes denaturation of sarcoplasmic proteins, which play a role in the emulsification and gelation properties of meat. This further reduces the WHC, particularly in processed meat products where these functional attributes are critical.

Postmortem pH decline is another key factor influenced by HS. Animals exposed to high heat may enter slaughter with altered muscle glycogen reserves due to hypermetabolic activity and reduced feed intake. This can lead to rapid glycolysis and a steep drop in pH soon after slaughter, resulting in protein denaturation and pale, soft, and exudative (PSE) meat, especially in pigs and poultry [9]. Conversely, prolonged HS may deplete glycogen to the point that postmortem acidification is incomplete, yielding dark, firm, and dry (DFD) meat with elevated ultimate pH values. Both outcomes lead to poor WHC and inferior eating quality.

Flavour development is intimately tied to lipid oxidation, amino acid degradation, and Maillard reactions during cooking. HS promotes oxidative stress, increasing the formation of ROS that initiate lipid peroxidation. This process produces aldehydes, ketones, and alcohols that contribute to rancid, metallic, or sour off-flavours [55]. Furthermore, HS reduces the availability of flavour precursors such as sulphur-containing amino acids, which limits the generation of umami-rich compounds upon cooking [53].

Changes in intramuscular fat composition further influence flavour. HS often leads to reduced PUFA content and elevated SFA proportions in muscle. PUFA oxidation produces flavourful compounds during cooking, so their reduction diminishes the depth of flavour in HS meat [56]. Additionally, a high SFA content may make fat deposits more solid and less palatable, impacting consumer satisfaction.

Colour, a vital visual cue for consumers, is highly sensitive to heat-induced oxidative stress. Meat coloration is determined by the redox state of myoglobin, a haem protein responsible for oxygen storage. In its oxygenated state (oxymyoglobin), it confers a bright red colour considered desirable in fresh meat. However, HS accelerates the oxidation of oxymyoglobin to metmyoglobin, giving meat an unappealing brown colour associated with spoilage [57]. Antioxidants like vitamin E normally help stabilize oxymyoglobin, but HS reduces their concentrations in muscle, further exacerbating discoloration [58].

The rate of discoloration under HS is accelerated by lower muscle pH, oxidative imbalance, and limited oxygen diffusion in damaged tissues. This rapid browning reduces display life and marketability in retail environments, where consumers rely heavily on appearance. Meat that turns brown prematurely is often discarded, contributing to economic losses across the supply chain.

Nevertheless, the severity of HS effects on meat quality is influenced by genetic, nutritional, and environmental factors. Some animal lines exhibit better thermotolerance due to elevated expression of heat shock proteins (e.g., HSP70), which help preserve cellular function and minimize oxidative damage [28]. Similarly, strategic feeding interventions using antioxidants such as vitamin E, selenium, alpha-lipoic acid, and polyphenols have shown promise in counteracting HS-induced biochemical damage and preserving flavour, tenderness, and shelf life [40].

In addition to nutritional and genetic strategies, post-harvest interventions like sous-vide cooking, vacuum packaging, and modified atmosphere storage help stabilize meat quality under HS. These methods reduce oxygen exposure, limit protein denaturation, and help preserve WHC and flavour profiles, particularly in meat from heat-exposed animals [55].

In summary, heat stress has a multifactorial impact on meat quality attributes, negatively influencing tenderness, juiciness, flavour, and colour through a combination of oxidative damage, protein denaturation, and metabolic disruption. While advances in nutritional, genetic, and processing interventions offer mitigation strategies, rising global temperatures remain a significant challenge for meat producers. A coordinated approach integrating animal selection, environmental control, and precise feeding is essential to safeguard meat quality and consumer satisfaction in the face of climate-driven HS.

Table 1 provides a detailed overview of how HS affects the nutritional composition of meat from monogastric animals. It summarizes the specific changes in proteins and amino acids, lipids and fatty acid profiles, vitamins and minerals, and overall meat quality attributes. The table also outlines the underlying mechanisms driving these changes and their implications for both meat quality and human health, with supporting references from key studies.

## 4. Human Health Implications

HS alters the nutrient profile of meat from monogastric animals, reducing essential proteins, fats, vitamins, and minerals vital for metabolism, muscle health, and immune function. These changes not only lower nutritional value but may also increase the risk of chronic conditions like cardiovascular disease, metabolic disorders, and inflammation, especially for populations relying heavily on meat. This section examines the health implications of consuming meat from heat-stressed animals, focusing on nutrient composition, bioavailability, and related risks.

### 4.1. Nutritional Quality of Meat and Dietary Impact

Meat from monogastric animals such as pigs and poultry is a vital source of high-quality macronutrients (proteins, lipids) and micronutrients (vitamins, minerals), playing a crucial role in human nutrition. It contributes essential amino acids, PUFAs, and bioavailable micronutrients like iron, zinc, and selenium, which support muscle repair, metabolic homeostasis, immune defence, and cognitive function. However, HS disrupts animal physiology and metabolism, significantly altering the nutritional composition of meat and reducing its dietary value and health benefits [59,68].

Heat-stressed meat typically shows decreased concentrations of high-quality proteins, essential amino acids, unsaturated fatty acids, and key vitamins and minerals. This degradation results from reduced feed intake, compromised intestinal absorption, and systemic oxidative stress, all of which disrupt nutrient assimilation and muscle deposition [30,69].

Total muscle protein content can decline by up to 15% under HS, with values falling from 23 g/100 g to as low as 19.5 g/100 g. This reduction is largely due to increased proteolytic activity, primarily through the ubiquitin–proteasome system and autophagy pathways, combined with impaired protein synthesis driven by suppressed insulin and IGF-1 signalling [3]. Such reductions compromise the dietary protein supply and can impair recovery and immunity, especially in children, the elderly, and protein-deficient populations.

The EAA profile is also modified. The concentrations of lysine, methionine, and threonine, critical for muscle anabolism, immunity, and neurotransmitter synthesis, can decrease by 15–25%. For example, the lysine content in pork may drop from 1.8 to 1.4 g/100 g, methionine from 0.6 to 0.45 g/100 g, and threonine from 1.0 to 0.8 g/100 g under HS [70]. These reductions lower the biological value of meat and limit its ability to fulfil amino acid requirements in humans, particularly when dietary diversity is low [71].

HS also induces profound shifts in muscle lipid composition. The levels of beneficial PUFAs, especially omega-3 fatty acids such as EPA and DHA, decline due to enhanced peroxidation, while SFAs tend to increase. The typical omega-6 to omega-3 ratio in healthy meat is ~4:1, but in HS meat this can rise to 10:1 or higher, significantly exceeding recommendations and promoting inflammatory responses when consumed regularly [72]. This reduction in PUFA content not only affects cardiovascular and cognitive health outcomes in consumers but also reduces the generation of favourable flavour compounds during cooking.

The micronutrient content also declines under HS. Fat-soluble vitamins like vitamins A, D, and E are sensitive to oxidative degradation. For example, vitamin E levels can fall by 30–50% in HS meat, weakening antioxidant defences and membrane stability. Similarly, vitamin A and vitamin D show reduced absorption and tissue retention due to impaired feed intake and stress-altered metabolism [73].

Essential trace elements, including selenium, zinc, and iron, also decline. Selenium, a cofactor for glutathione peroxidase, is particularly depleted under oxidative stress. Zinc levels drop due to impaired absorption and increased excretion, limiting its roles in DNA synthesis, antioxidant function, and immune defence. Iron loss is equally critical, as it reduces haemoglobin and myoglobin content, impairing oxygen transport and energy metabolism. These losses are especially detrimental to pregnant women, children, and the elderly, for whom these micronutrients are essential [6,59].

In summary, HS significantly compromises the dietary quality of meat through multi-nutrient degradation. Quantitative reductions in proteins, EAAs, PUFAs, and key micronutrients like selenium and vitamin E reduce the nutritional density of meat, particularly in populations already at risk for dietary insufficiencies. From a public health perspective, increased consumption of heat-stressed meat could exacerbate nutrient gaps and increase susceptibility to metabolic disorders.

### 4.2. Potential Health Risks

The altered nutritional composition of meat under HS poses multiple health risks, primarily due to increased saturated fat content, reduced omega-3 fatty acids, and diminished antioxidant levels such as vitamin E and selenium. These biochemical changes carry systemic implications for cardiovascular, metabolic, immune, and neurological health. In populations where meat is a primary nutrient source, regular consumption of heat-stressed meat may contribute to a higher incidence of diet-related diseases.

One major concern is the altered fatty acid composition. HS reduces the concentration of beneficial PUFAs, particularly omega-3s like EPA and DHA, due to oxidative degradation and disrupted enzymatic desaturation pathways [72]. Simultaneously, levels of SFAs increase, resulting in a higher SFA:PUFA ratio and a skewed omega-6 to omega-3 ratio. Ideally, this ratio should remain around 4:1 for optimal health, but in heat-stressed meat, it may exceed 10:1 [74]. This imbalance promotes inflammation and has been linked to chronic illnesses such as cardiovascular disease, arthritis, and inflammatory bowel disease [75].

Elevated SFA intake also contributes to higher LDL cholesterol levels, endothelial dysfunction, and plaque formation in arteries, key risk factors for heart disease and stroke. Additionally, excessive saturated fat stimulates hepatic lipogenesis and can lead to non-alcoholic fatty liver disease (NAFLD) and insulin resistance [75].

Neurologically, the loss of DHA, a primary structural component of brain membranes, impairs synaptic function, mood regulation, and memory formation. Reduced DHA intake has been associated with increased rates of depression, cognitive decline, and neurodegenerative conditions like Alzheimer’s disease and Parkinson’s disease [76].

A skewed fatty acid profile also alters immune regulation. Omega-3 fatty acids are precursors to anti-inflammatory lipid mediators (resolvins, protectins), whereas omega-6 derivatives promote pro-inflammatory cytokines like IL-6 and TNF-α. Their imbalance heightens the risk of autoimmune conditions, chronic inflammation, and delayed tissue repair [77].

Beyond lipid alterations, HS reduces the antioxidant content of meat. Vitamin E is depleted due to increased lipid peroxidation, weakening cellular defence against free radicals. This raises the risk of oxidative stress-mediated conditions, including atherosclerosis, cancer, and neuronal damage [74]. Lipid peroxidation also generates reactive aldehydes and other toxic compounds during cooking, further amplifying cellular damage upon consumption.

Selenium, a cofactor in the glutathione peroxidase system, is another critical antioxidant compromised under HS. Its deficiency reduces the body’s capacity to neutralize hydrogen peroxide and lipid peroxides, exacerbating oxidative injury and immune dysregulation [78].

The combination of reduced antioxidant availability and increased oxidative stress leads to DNA damage, impaired mitochondrial function, and disrupted cellular signalling. These processes underlie the progression of cancer, type 2 diabetes, and neurodegenerative conditions.

The systemic health implications of consuming meat from heat-stressed animals stem from both compositional nutrient losses and the formation of pro-inflammatory and pro-oxidative compounds. Although experimental and animal model studies compellingly demonstrate reduced nutrient density and increased levels of harmful compounds, direct epidemiological evidence linking these alterations to specific chronic health outcomes in humans remains limited [6]. Consequently, the translation of these findings into quantifiable human dietary risks necessitates cautious interpretation. Addressing these challenges through improved animal management, tailored dietary interventions, and enhanced consumer education is essential to safeguarding public health. Future research should prioritize large-scale, prospective epidemiological studies that integrate biomarker assessments and rigorously control for confounding factors to strengthen the causal interpretation of the observed experimental effects [32,79].

### 4.3. Dietary Recommendations

Given the nutritional challenges posed by heat-stressed meat, it is crucial to adopt dietary strategies that compensate for the altered nutrient profiles and mitigate the associated health risks. The reduction in essential nutrients such as high-quality proteins, omega-3 fatty acids, and antioxidants, combined with an increase in saturated fats, necessitates targeted adjustments in dietary patterns and food choices. Effective dietary interventions can help balance nutrient intake, improve overall health outcomes, and reduce the long-term risks of chronic diseases linked to poor meat quality. This section outlines key dietary recommendations to address the nutritional deficits of heat-stressed meat and improve public health outcomes.

The reduction in total protein content and essential amino acids in heat-stressed meat requires strategies to maintain adequate protein intake and ensure a balanced amino acid profile. Since heat-stressed meat is often deficient in essential amino acids like lysine, methionine, and threonine, combining it with other protein sources can help restore the balance of essential nutrients.

Diversifying protein sources is an effective strategy to compensate for the lower protein quality of heat-stressed meat. A mixed diet that includes plant-based proteins such as legumes, lentils, soy, and nuts, along with other animal proteins like fish, eggs, and dairy, can help balance the amino acid profile and improve overall protein intake [66]. For example, legumes are naturally rich in lysine but low in methionine, whereas cereals provide methionine but are low in lysine. Combining these complementary proteins helps create a more complete amino acid profile, ensuring that essential amino acid requirements are met even when the protein quality of meat is compromised.

In populations where meat is a primary protein source, protein supplementation can be a practical solution to increase the availability of essential amino acids. High-quality plant-based or whey protein powders can provide a concentrated source of amino acids, enhancing muscle protein synthesis and metabolic function. Protein hydrolysates, which contain pre-digested peptides, can further improve protein absorption and utilization by bypassing some digestive processes, making the amino acids more readily available to support muscle repair and growth [80].

Another promising approach is the biofortification of meat through the dietary modification of animal feed. Enriching animal diets with essential amino acids or high-quality protein sources can enhance the amino acid content of muscle tissue, even under HS conditions. For instance, feeding monogastric animals diets enriched with lysine and methionine has been shown to improve muscle protein composition and increase the overall protein quality of meat [3]. Biofortification not only enhances the nutritional value of meat but also supports better muscle development and growth in animals, thereby improving the quality and health benefits of meat for human consumption.

The increased saturated fat content and reduced omega-3 concentration in heat-stressed meat create an unhealthy lipid profile that can increase the risk of cardiovascular and metabolic diseases. Adjusting dietary fat intake by increasing omega-3 consumption and reducing saturated fat intake is critical for maintaining the lipid balance and improving overall health outcomes.

To counteract the reduction in omega-3 fatty acids in heat-stressed meat, dietary recommendations should emphasize the consumption of omega-3-rich foods. Fatty fish such as salmon, mackerel, and sardines are excellent sources of EPA and DHA, the two most biologically active forms of omega-3 fatty acids [81]. Regular intake of EPA and DHA from fish and other marine sources has been shown to lower triglyceride levels, reduce inflammation, and improve cognitive function. The anti-inflammatory properties of omega-3 fatty acids also contribute to better cardiovascular health by reducing blood pressure and improving vascular function [82].

For vegetarians and populations with limited access to seafood, plant-based omega-3 sources such as flaxseed oil, chia seeds, and walnuts are valuable dietary options. These foods provide alpha-linolenic acid (ALA; 18:3n-3), a precursor to EPA and DHA. However, the conversion of ALA to EPA and DHA in the human body is relatively inefficient, with conversion rates estimated at less than 10% [83]. To overcome this limitation, fortified products containing algal-oil-based DHA can help meet omega-3 requirements more effectively. Algal oil is a sustainable and vegetarian-friendly source of DHA that bypasses the need for metabolic conversion, making it an efficient option for increasing omega-3 intake in plant-based diets.

Reducing saturated fat intake is another key strategy to balance lipid metabolism and improve cardiovascular health. High levels of saturated fats in heat-stressed meat increase LDL cholesterol, which promotes atherosclerosis and increases the risk of heart disease. Substituting lean meats such as poultry and fish for heat-stressed pork or beef can help lower saturated fat intake while maintaining protein quality. Cooking methods such as grilling, baking, or broiling instead of frying can further reduce saturated fat consumption and improve the overall fat profile of the diet. A lower saturated fat intake not only reduces cardiovascular risk but also supports metabolic health by improving insulin sensitivity and reducing inflammation [84].

The fortification of animal feed with omega-3-rich ingredients provides another effective strategy to improve the fatty acid profile of meat from heat-stressed animals. Feeding monogastric animals flaxseed, fish oil, or microalgae increases the omega-3 content of their muscle tissues, enhancing the nutritional quality of the meat produced. Studies have shown that dietary supplementation with fish oil or algae can increase muscle EPA and DHA concentrations, leading to meat with improved anti-inflammatory and cardioprotective properties [62,85,86]. Strategies to increase microalga nutrient bioavailability have also been developed [87,88]. This approach not only improves human health outcomes but also enhances the market value of meat products by providing consumers with healthier options.

The reduced retention of vitamin E, selenium, and zinc in heat-stressed meat lowers the antioxidant potential of the diet and increases the risk of oxidative stress. Improving antioxidant intake through dietary and supplementation strategies is essential for maintaining cellular health and reducing inflammation.

Increasing the intake of antioxidant-rich foods is an effective strategy for enhancing the body’s ability to neutralize the oxidative stress caused by heat-stressed meat. A diet rich in fruits, vegetables, and whole grains provides essential antioxidants such as vitamins C and E, polyphenols, and flavonoids, which help counteract the damaging effects of ROS. Leafy greens such as spinach and kale, as well as berries, nuts, and seeds, are particularly rich in antioxidants and should be incorporated into daily diets to strengthen the body’s defence mechanisms against oxidative damage [8]. The polyphenols and flavonoids found in fruits and vegetables have been shown to reduce inflammation, protect vascular health, and improve immune function by scavenging free radicals and enhancing cellular repair processes.

In populations where heat-stressed meat is a major dietary staple, supplementation with vitamin E and selenium may be necessary to maintain adequate antioxidant defences. Vitamin E is a fat-soluble antioxidant that protects cell membranes from lipid peroxidation by interrupting the chain reaction of free radical damage. Reduced levels of vitamin E in heat-stressed meat lower the antioxidant capacity of the diet, increasing the body’s vulnerability to oxidative stress and inflammation. Selenium, on the other hand, is essential for the function of glutathione peroxidase, an enzyme that neutralizes hydrogen peroxide and reduces oxidative stress within cells. A low selenium intake compromises immune function and increases the risk of inflammatory and metabolic disorders. Therefore, targeted supplementation with vitamin E and selenium can help restore the antioxidant balance and reduce the health risks associated with heat-stressed meat consumption [66].

Another promising approach to improving the antioxidant content in meat is the biofortification of animal feed with micronutrients such as selenium and vitamin E. Fortifying the diets of monogastric animals with these nutrients enhances their retention in muscle tissue, thereby improving the oxidative stability of the meat produced. Studies have shown that supplementing poultry feed with selenium and vitamin E increases their concentration in muscle tissues, enhancing the antioxidant potential of the resulting meat products [89]. Improved oxidative stability not only increases the shelf life of meat but also enhances its nutritional value by reducing the extent of lipid oxidation and improving sensory attributes such as flavour and colour. Biofortification represents a cost-effective strategy for improving the nutritional profile of meat and supporting better health outcomes in populations that rely heavily on meat as a primary protein source [90].

Educating consumers about the nutritional consequences of heat-stressed meat and providing guidance on dietary adjustments is essential for improving health outcomes. Public awareness about the effects of HS on meat quality, including reduced protein content, altered fatty acid profiles, and lower levels of essential vitamins and minerals, can empower consumers to make healthier dietary choices. Understanding these nutritional changes allows consumers to adjust their diets to maintain balanced nutrient intake and reduce the health risks associated with heat-stressed meat.

Promoting balanced diets through public health campaigns is a key strategy for mitigating the health risks associated with heat-stressed meat. Public health messages should emphasize the importance of consuming a varied and balanced diet that includes a range of protein sources, omega-3-rich foods, and antioxidant-rich fruits and vegetables. Encouraging the consumption of both plant-based and animal-derived proteins ensures that essential amino acid requirements are met, even when the protein quality of meat is compromised. Increased intake of omega-3 fatty acids from fatty fish, walnuts, and flaxseeds can help counteract the higher saturated fat content in heat-stressed meat, while antioxidant-rich foods such as leafy greens, berries, and nuts can strengthen the body’s ability to manage oxidative stress [91].

Labelling and transparency in food production can further support consumer decision making. Providing clear information about the nutritional composition of meat products, including omega-3 content, saturated fat levels, and antioxidant capacity, allows consumers to make informed choices. Labelling that highlights key nutrient information can guide consumers toward healthier purchasing decisions and increase demand for higher-quality meat products. Transparency regarding the production process, including whether animals were raised under HS conditions or fed fortified diets, can also improve consumer trust and influence purchasing behaviour [92].

Government and industry support are critical in promoting dietary improvements and reducing the nutritional impact of HS. Policymakers and food producers can support sustainable farming practices that minimize HS in livestock through improved housing, ventilation systems, and cooling strategies. Promoting the use of fortified animal feed enriched with essential amino acids, omega-3 fatty acids, and antioxidants can enhance the nutritional quality of meat even under HS conditions. Government incentives for sustainable agriculture and food production practices can encourage industry-wide adoption of these strategies, ultimately improving the nutritional profile of meat products and supporting public health.

Table 2 summarises how HS-induced changes in meat affect human nutrition, outlines the resulting health risks, and suggests dietary interventions to help mitigate these adverse effects.

## 5. Nutritional Mitigation Strategies

HS adversely affects the nutritional composition of meat from monogastric animals by disrupting metabolic processes, impairing muscle growth, and altering nutrient absorption. These changes increase oxidative stress, reduce production efficiency, and diminish meat quality for human consumption. Targeted nutritional interventions can mitigate these metabolic disruptions and oxidative damage by optimizing animal diets and incorporating specific bioactive compounds, ultimately preserving meat quality and improving animal welfare [95,96]. The key strategies involve antioxidant supplementation, osmolyte supplementation, and dietary energy and protein optimization.

### 5.1. Antioxidant Supplementation

Under conditions of HS, animals experience an imbalance between the production of ROS and the capacity of their endogenous antioxidant defences. ROS, such as superoxide anion, hydroxyl radicals, and hydrogen peroxide, are normal by-products of cellular metabolism. However, when the metabolic rate increases under HS, ROS production exceeds the neutralizing capacity of enzymatic antioxidants like superoxide dismutase, catalase, and glutathione peroxidase, as well as non-enzymatic antioxidants such as vitamin E, vitamin C, and glutathione. This leads to oxidative damage to lipids, proteins, and DNA, impairing muscle growth and nutrient absorption while compromising meat quality [59].

Vitamin E is a fat-soluble antioxidant that plays a critical role in protecting cell membranes from lipid peroxidation, a process that forms damaging by-products such as MDA. In heat-stressed animals, the depletion of natural vitamin E reserves reduces the muscle’s ability to resist oxidative damage. Supplementation with vitamin E at levels typically ranging between 100 and 400 IU/kg of feed has been shown to maintain membrane integrity, improve meat colour stability, reduce drip loss, and enhance tenderness and flavour [8,65].

Selenium, a trace mineral, functions as a cofactor for selenoproteins, including glutathione peroxidase, which reduces hydrogen peroxide and lipid peroxides to less harmful molecules. Under HS, the increased production of ROS raises the demand for selenium, and its supplementation (0.1 to 1 mg/kg diet) enhances the activity of glutathione peroxidase, thereby reducing oxidative damage and improving muscle function [59,65]. Selenium and vitamin E work synergistically; while vitamin E scavenges lipid peroxyl radicals, selenium-based enzymes eliminate hydrogen peroxide and other peroxides, preventing the propagation of oxidative damage.

Additionally, the polyphenols found in sources such as green tea, grape seed extract, rosemary, olive leaf extract, and algae [85,86], offer strong antioxidant and anti-inflammatory properties. They act by directly scavenging ROS, chelating metal ions that catalyse oxidative reactions, and upregulating endogenous antioxidant enzymes, which together reduce lipid oxidation and improve the sensory attributes and shelf life of meat [80]. Antioxidant supplementation thus improves meat quality by increasing free radical scavenging, enhancing endogenous defence systems, stabilizing cell membranes, reducing muscle protein degradation, and improving sensory attributes. However, it was noted that excessive antioxidant supplementation may lead to pro-oxidant effects under certain conditions.

### 5.2. Osmolyte Supplementation

Osmolyte supplementation offers another effective strategy to mitigate the detrimental effects of HS. Osmolytes are small organic molecules that maintain cellular osmoregulation, protein stabilization, and metabolic balance. HS causes cellular dehydration, ionic imbalances, and increased metabolic heat production, which impair nutrient absorption, reduce muscle protein synthesis, and elevate oxidative damage.

Betaine (trimethylglycine) is an osmolyte that functions as an osmoprotectant by regulating osmotic balance and stabilizing cellular structures. It also acts as a methyl donor in one-carbon metabolism, supporting the synthesis of key compounds necessary for muscle metabolism and protein synthesis [97]. During HS, reduced feed intake limits the availability of methyl donors such as methionine and choline; betaine supplementation (0.5–2 g/kg of feed) helps to compensate for this deficit, enhancing muscle growth and stimulating lipid oxidation while activating the mTOR pathway [98].

Taurine, a sulphur-containing amino acid, functions as an osmolyte, antioxidant, and modulator of calcium homeostasis. Found in high concentrations in muscle tissue, taurine regulates ion balance, stabilizes cell membranes, and supports efficient muscle contraction [99]. Under HS, taurine levels decline due to increased cellular turnover and oxidative damage; its supplementation (0.1–0.5 g/kg of feed) helps restore these levels, stabilizing calcium channels and improving muscle contraction efficiency while reducing fatigue. Additionally, taurine enhances the activity of endogenous antioxidant enzymes such as superoxide dismutase and glutathione peroxidase, reducing lipid peroxidation and protecting muscle proteins from degradation [100]. The combined effects of betaine and taurine help maintain cellular hydration by balancing osmotic pressure and preserving muscle cell integrity, elasticity, and water-holding capacity, which in turn enhances meat texture and juiciness. However, excessive osmolyte supplementation may lead to imbalances that could disrupt normal metabolic processes.

### 5.3. Dietary Energy and Protein Optimization

HS naturally reduces feed intake in monogastric animals as they attempt to lower metabolic heat production, but this reduction results in an energy and nutrient deficit that compromises muscle growth and protein synthesis. To address these challenges, optimizing the energy and protein content of animal diets is essential. Increasing dietary energy density by incorporating highly digestible fats such as soybean oil, poultry fat, fish oil, and flaxseed oil provides a calorie-dense energy source with a lower heat increment compared to carbohydrates or proteins, ensuring that animals meet their energy requirements even with reduced feed intake [101]. While highly digestible carbohydrates can also increase energy availability, their higher heat increment necessitates a balanced inclusion with fats to avoid exacerbating HS [102].

In addition to energy density, optimizing dietary protein is crucial because HS not only reduces muscle protein synthesis but also increases muscle protein degradation, partly due to elevated cortisol levels. Increasing the overall protein content of the diet by 5% to 10% under HS conditions helps compensate for reduced essential amino acid intake; however, the focus must also be on improving the amino acid profile [103]. Essential amino acids such as lysine, methionine, and threonine are critical for muscle protein synthesis, growth, and repair. Lysine, often the first limiting amino acid in monogastric diets, is particularly important, and supplementing with crystalline lysine can enhance muscle growth even when feed intake is low. Methionine and threonine support the synthesis of structural proteins and immune-related molecules, which are essential for maintaining muscle integrity and immune function under stress [66]. Protein hydrolysates, which are composed of pre-digested peptides and free amino acids, offer a readily absorbed source of nutrients that support muscle repair and growth. Balancing amino acids with appropriate fat sources also plays a critical role in maintaining meat quality. Incorporating unsaturated fats such as fish oil and flaxseed oil not only improves the nutritional profile of the meat by enhancing the omega-3 to omega-6 ratio but also improves oxidative stability and shelf life. Adjustments such as increasing the dietary fat content by 3% to 5% during periods of high HS and boosting essential amino acids by 10% to 20% above normal requirements help maintain muscle integrity and the metabolic balance despite reduced feed intake [103].

### 5.4. Economic and Practical Implications

Beyond the biochemical and nutritional impacts, HS presents significant economic and practical challenges for the meat industry. Reduced feed efficiency, impaired growth rates, increased mortality, and compromised meat quality contribute to substantial financial losses in swine and poultry production. Implementing nutritional mitigation strategies, such as antioxidant supplementation (e.g., vitamin E, selenium) and osmolyte supplementation (e.g., betaine, taurine), has shown promise in improving production efficiency and meat quality under HS conditions. For example, betaine supplementation has been linked to improved feed conversion ratios and enhanced muscle protein retention, leading to better carcass quality and higher market value [104].

Practical recommendations to mitigate HS in production systems include optimizing ventilation, providing shade and cooling systems, and adjusting feeding schedules to avoid peak heat periods [15,105]. Nutritional strategies should focus on balancing energy sources, increasing dietary fat content to reduce metabolic heat production, and enhancing antioxidant intake to combat oxidative stress. Policymakers could support these interventions by funding research on HS resilience and providing incentives for adopting climate-adaptive management practices. Strengthening industry resilience to HS will help maintain production efficiency, improve meat quality, and ensure the economic sustainability of monogastric production systems in the face of rising global temperatures.

Incorporating nutritional and environmental mitigation strategies to combat heat stress involves additional upfront costs, such as investments in dietary supplements (e.g., vitamin E, selenium, betaine, and taurine) and modifications to housing and cooling systems. However, these costs can be counterbalanced by improved production efficiency, including enhanced meat quality, lower feed conversion ratios, and reduced mortality rates [106]. In regions where heat stress significantly impairs livestock performance, the economic losses associated with decreased productivity are substantial; thus, adopting these interventions offers a potentially favourable return on investment. Moreover, governmental policies and industry subsidies that promote climate-resilient livestock practices can further support the economic viability of these measures [107].

## 6. Conclusions and Future Perspectives

Rising global temperatures and the increasing frequency of heatwaves have made HS a critical challenge for monogastric livestock production. HS impairs feed intake, induces oxidative stress, disrupts endocrine function, and alters nutrient metabolism, ultimately compromising both the nutritional composition and sensory quality of meat. This review has shown that HS reduces the total muscle protein content by up to 15%, shifts amino acid profiles, increases SFA proportions, and depletes essential micronutrients such as vitamin E, selenium, and iron. These changes not only reduce the nutritional value and shelf life of meat but also pose significant risks to human health, particularly in populations with limited dietary diversity or high dependency on meat for essential nutrients.

From a quality perspective, HS leads to meat that is paler, less juicy, more susceptible to oxidative spoilage, and nutritionally inferior. The risk of chronic diseases such as cardiovascular disease, diabetes, and neurodegeneration may be amplified through regular consumption of heat-stressed meat due to elevated SFA levels, reduced omega-3 intake, and diminished antioxidant content.

Mitigation strategies are urgently needed and should be multi-tiered. Nutritional approaches such as antioxidant and osmolyte supplementation (e.g., vitamin E, selenium, betaine) have shown potential in preserving muscle integrity and reducing oxidative damage by up to 40%. Genetic selection for heat-resilient breeds that overexpress heat shock proteins (e.g., HSP70) also holds promise, as does environmental management, improved ventilation, shading, and climate-adaptive housing systems.

Future research should prioritize high-resolution molecular profiling (proteomics, metabolomics, transcriptomics) to identify early biomarkers of HS in muscle tissue. Additionally, understanding species- and breed-specific responses to HS will help tailor interventions more effectively. More studies are also needed on the long-term health effects of consuming heat-stressed meat, especially in nutritionally vulnerable populations.

## Figures and Tables

**Table 1:** Summary of the effects of heat stress on meat nutritional composition. Abbreviations: EAA, Essential Amino Acid; HPA, Hypothalamic–Pituitary–Adrenal; MDA, Malondialdehyde; MUFA, Monounsaturated Fatty Acid; NEAA, Non-Essential Amino Acid; PUFA, Polyunsaturated Fatty Acid; SFA, Saturated Fatty Acid; UPS, Ubiquitin–Proteasome System.

Component	Changes Under Heat Stress	Mechanisms	Implications for Meat Quality and Human Health	References
Proteins and Amino Acids	Reduced total muscle protein content Lower concentrations of lysine, methionine, threonine Skewed essential to non-essential amino acid ratio Increased levels of glutamine and alanine	Reduced feed intake and digestive efficiency Activation of cortisol and suppression of insulin and IGF-1 Impaired mTOR pathway and increased proteolysis via UPS and autophagy	Reduced protein quality and bioavailability Impaired muscle repair and immune function Increased risk of protein deficiency in vulnerable populations	[7,59,60,61]
Lipids and Fatty Acid Profiles	Increased saturated fatty acids (SFAs) Reduced polyunsaturated fatty acids (PUFAs), especially omega-3 Elevated omega-6/omega-3 ratio Reduced monounsaturated fatty acids (MUFAs)	Elevated oxidative stress causing lipid peroxidation Downregulation of stearoyl-CoA desaturase (SCD) Selective loss of omega-3 fatty acids	Lower cardiovascular and anti-inflammatory value of meat Reduced flavour, tenderness, and shelf life Elevated health risk from higher SFA intake	[59,62,63,64]
Vitamins and Minerals	Lower levels of vitamins A, D, E, K, and B-complex Depletion of vitamin C, selenium, zinc, iron, calcium, and magnesium Impaired antioxidant capacity and mineral retention	Reduced feed intake and intestinal absorption Elevated oxidative stress increasing vitamin and mineral demand Increased urinary excretion and muscle depletion	Nutrient losses decrease dietary value Increased susceptibility to oxidative spoilage Higher risk of micronutrient deficiency in consumers	[10,59,65,66]
Meat Quality Attributes	Decreased tenderness and juiciness Increased lipid oxidation and drip loss Greater myoglobin oxidation causes colour deterioration Reduced flavour depth due to PUFA loss	Cortisol-induced proteolysis and oxidative protein damage Accelerated myoglobin oxidation to metmyoglobin Oxidative degradation of lipids and pigments	Reduced consumer appeal and economic value Shortened shelf life and greater spoilage Negative perception due to colour and texture degradation	[8,59,61,67]

**Table 2:** Summary of the potential effects of heat-stressed meat on human health. Abbreviations: EAA, Essential Amino Acid; Omega-3, Omega-3 Fatty Acid; PUFA, Polyunsaturated Fatty Acid; SFA, Saturated Fatty Acid.

Aspect	Key Observations/Mechanisms	Health Implications	Recommendations/Interventions	References
Nutritional Quality and Dietary Impact	Heat stress in animals leads to lower high-quality proteins, essential amino acids (e.g., lysine, methionine, and threonine), beneficial fatty acids (decline in omega-3; rise in saturated fats and omega-6/omega-3 imbalance), and reduced levels of key vitamins and minerals.	Reduced nutrient density in meat may result in lower overall protein quality and micronutrient deficiencies, particularly for populations relying heavily on meat for essential nutrients.	Diversify protein sources (e.g., combined with plant proteins) and adjust dietary plans to offset the nutrient deficits.	[10,59,81]
Potential Health Risks	The altered meat composition (increased saturated fats, decreased omega-3 fatty acids, and lower antioxidant content) promotes a pro-inflammatory state and oxidative stress.	Elevated risks for cardiovascular disease, metabolic disorders, chronic inflammation, and potentially adverse cognitive and immune effects.	Shift toward leaner meats, balance fat intake, and include foods rich in omega-3 and antioxidants to help reduce risks.	[62,81,93,94]
Dietary Recommendations	Strategies include incorporating omega-3-rich foods (e.g., fatty fish, walnuts, flaxseeds), increasing antioxidants (e.g., vitamin E, selenium), and promoting dietary diversification.	Improving dietary balance can mitigate the health risks of consuming heat-stressed meat and support overall nutritional well-being.	Public health education, improved nutritional labelling, and targeted supplementation or feed strategies to enhance meat quality.	[8,66,84]

## Data Availability

No new data were created or analysed in this study. Data sharing is not applicable to this article.
